# Radiomics of Vascular Structures in Pulmonary Ground-glass Nodules: A Predictor of Invasiveness

**DOI:** 10.2174/0115734056385352250410053810

**Published:** 2025-04-21

**Authors:** Wuling Wang, Xuan Qi, Yongsheng He, Hongkai Yang, Dong Qi, Zhen Tang, Qiong Chen

**Affiliations:** 1 Department of Radiology, Ma'anshan People's Hospital, Ma'anshan 243000, China; 2 Ma'anshan Key Laboratory for Medical Image Modeling and Intelligent Analysis, Maanshan 243099, China; 3 Ma'anshan Clinical College, Anhui Medical University, Hefei 230032, China; 4 School of Computer Science, Anhui University of Technology, Maanshan 243032, China; 5 Department of Radiology, Dahua Hospital of Xuhui District, Shanghai 200231, China

**Keywords:** Ground-glass nodule, Vascular structure, Invasiveness, Radiomics, Logistic regression, Support vector machine

## Abstract

**Objective:**

The global incidence of lung cancer highlights the need for improved assessment of nodule characteristics to enhance early detection of lung adenocarcinoma presenting as ground-glass nodules (GGNs). This study investigated the applicability of radiomics features of vascular structures within GGNs for predicting invasiveness of GGNs.

**Methods:**

In total, 165 pathologically confirmed pulmonary GGNs were retrospectively analyzed. The nodules were classified into preinvasive and invasive groups and randomly categorized into training and validation sets in a 7:3 ratio. Four models were constructed and evaluated: radiomics-GGN, radiomics-vascular, clinical-radiomics-GGN, and clinical-radiomics-vascular. The predictive performance of these models was assessed using receiver operating characteristic curves, decision curve analysis, calibration curves, and DeLong’s test.

**Results:**

Significant differences and density were observed between the preinvasive and invasive groups in terms of age, nodule length, average diameter, morphology, lobulation sign (*P* = 0.006, 0.038, 0.046, 0.049, 0.002 and0.008 respectively). In the radiomics-GGN model, the support vector machine (SVM) approach outperformed logistic regression (LR), achieving an area under the curve (AUC) of 0.958 in the training set and 0.763 in the validation set. Similarly, in the radiomics-vascular model, the SVM approach outperformed LR. Furthermore, the clinical-radiomics-vascular model demonstrated superior predictive performance compared with the clinical-radiomics-GGN model, with an AUC of 0.918 in the training set and 0.864 in the validation set. DeLong’s test indicated significant differences in predicting the invasiveness of pulmonary nodules between the clinical-radiomics-vascular model and the clinical-radiomics-GGN model, both in the training and validation sets (*P* < 0.01).

**Conclusion:**

The radiomics models based on internal vascular structures of GGNs outperformed those based on GGNs alone, suggesting that incorporating vascular radiomics analysis can improve the noninvasive assessment of GGN invasiveness, thereby aiding in clinical decision-making and guiding biopsy selection and treatment planning.

## 1 INTRODUCTION

Although the global incidence of lung cancer varies, it remains the leading cause of cancer-related death in men and the second most common cause of cancer mortality in women, following breast cancer [1]. Ground-glass nodules (GGNs), commonly considered markers of early-stage lung adenocarcinoma, are increasingly detected due to the widespread use of low-dose computed tomography (CT) and artificial intelligence-based diagnostic systems in routine screenings [2]. GGNs persisting for more than 3 months have a higher likelihood of malignancy than solid nodules, with 63.0%–95.5% confirmed as lung cancer [3-5]. According to the World Health Organization (WHO) Classification of Thoracic Tumours (5^th^ edition), lung adenocarcinomas are categorized as minimally invasive adenocarcinoma (MIA) and invasive adenocarcinoma (IA), whereas atypical adenomatous hyperplasia (AAH) and adenocarcinoma *in situ* (AIS) have been reclassified as precursor glandular lesions (PGLs) [6]. Patients with early-stage lung adenocarcinoma have shown a postoperative 5-year survival rate of 100% [7], whereas those with IA have shown a survival rate of 90.0%–99.1% [8, 9]. Wang *et al.* [10] reported that mixed GGNs (mGGNs) larger than 10 mm showed no recurrence or metastasis after 5 years. Accurate assessment of GGN invasiveness is crucial for appropriate treatment selection, timely intervention, and overtreatment prevention.

Currently, radiologists assess GGN invasiveness using CT features, such as diameter, density, lobulation, and spiculation; however, these indicators are highly subjective [6]. Radiomics is an emerging technology that utilizes machine vision to noninvasively extract comprehensive tumor biological information and transform it into quantitative features of diagnostic value [11]. Several studies, including those by Weng *et al.* [12] and Lv *et al.* [13], have developed radiomics models to differentiate IA from other GGN subtypes. IA and MIA often exhibit complex, irregular vascular structures; hence, vascular features within GGNs are critical indicators of malignancy and invasiveness. Gao *et al.* [14] classified GGN vascular features into four types: type I, vessels along the periphery; type II, vessels passing through without alteration; type III, twisted, dilated, or bent vessels; and type IV, vessels forming more intricate structures. However, owing to the typically delicate nature of internal vessels within GGNs, no prior studies have conducted radiomics analyses specifically targeting these vascular structures. Therefore, this study aimed to develop predictive models integrating clinical imaging features, radiomics features of vascular structures within GGNs, and clinical data to enhance the assessment of nodule invasiveness.

## 2 MATERIALS AND METHODS

### 2.1 Study Population

**Fig. (1):**
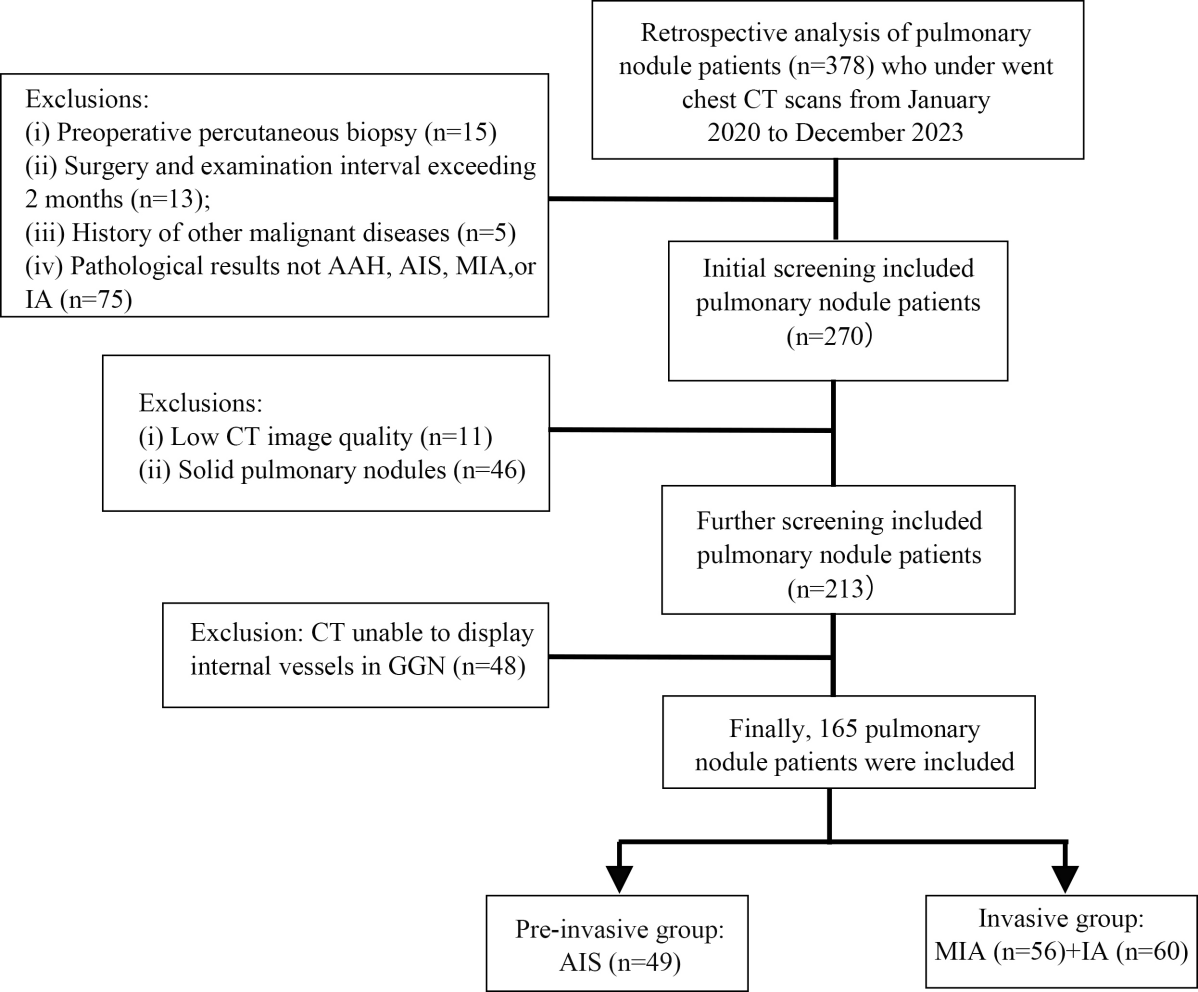
Sample enrollment, screening, and grouping flowchart.

This retrospective study analyzed patients with primary lung adenocarcinoma who had undergone chest CT at Ma'anshan City People’s Hospital between January, 2020 and December, 2023 and showed confirmed pathological results. Clinical data and preoperative CT images were collected. The inclusion criteria were as follows: (i) no neoadjuvant Therapy before surgery; (ii) surgery performed within 2 months of routine chest CT; (iii) nodules classified as pure GGNs (pGGNs) or mGGNs; (iv) CT images with sufficient quality to visualize vascular structures within GGNs; and (v) pathological confirmation as AAH, AIS, MIA, or IA. The exclusion criteria were as follows: (i) prior biopsy, thoracic surgery, or antitumor treatment before CT; (ii) history of other malignant diseases; (iii) low-quality CT images preventing accurate lesion delineation; (iv) poor visualization of vascular structures within GGNs; and (v) comorbid pulmonary fibrosis or emphysema (Fig. **1**). This study was approved by the ethics committee of our institution (approval no.: 2022012013), and the requirement for written informed consent was waived due to its retrospective design.

### 2.2 CT Examination

Noncontrast-enhanced CT was performed using a GE LightSpeed VCT 64 scanner and SOMATOM Force CT scanner. The patients were positioned supine, and scanning was conducted at the end of inspiration, covering the area from the thoracic inlet to the lung base. The scanning parameters were as follows: slice thickness, 5 mm; interslice gap, 5 mm; matrix size, 512 × 512; tube voltage, 120 kV; tube current, 150 or 200 mA; and reconstruction slice thickness, 1.0 mm.

### 2.3 Image Postprocessing and Analysis

#### 2.3.1 CT Image Postprocessing

All CT images were processed and analyzed by two physicians with 9 and 10 years of experience in chest imaging for disease diagnosis, respectively. Both physicians were blinded to the patients’ clinical and pathological information. Image interpretation was conducted in the lung window mode using an imaging diagnostic system, with a window width of 1500 HU and a window level of −600 HU. The recorded imaging characteristics of the nodules included location, long and short, average diameters, average CT value, morphology, lobulation, spiculation, pleural retraction, and density type. In cases of disagreement, a consensus was reached through discussion. Image postprocessing was performed using a Siemens Syngo. *via* workstation, primarily employing three-dimensional postprocessing and orthogonal analysis to obtain images displaying the maximum cross-section of vascular structures within pulmonary GGNs. The images were saved in DICOM format.

#### 2.3.2 Radiomics Analysis

**Fig. (2):**
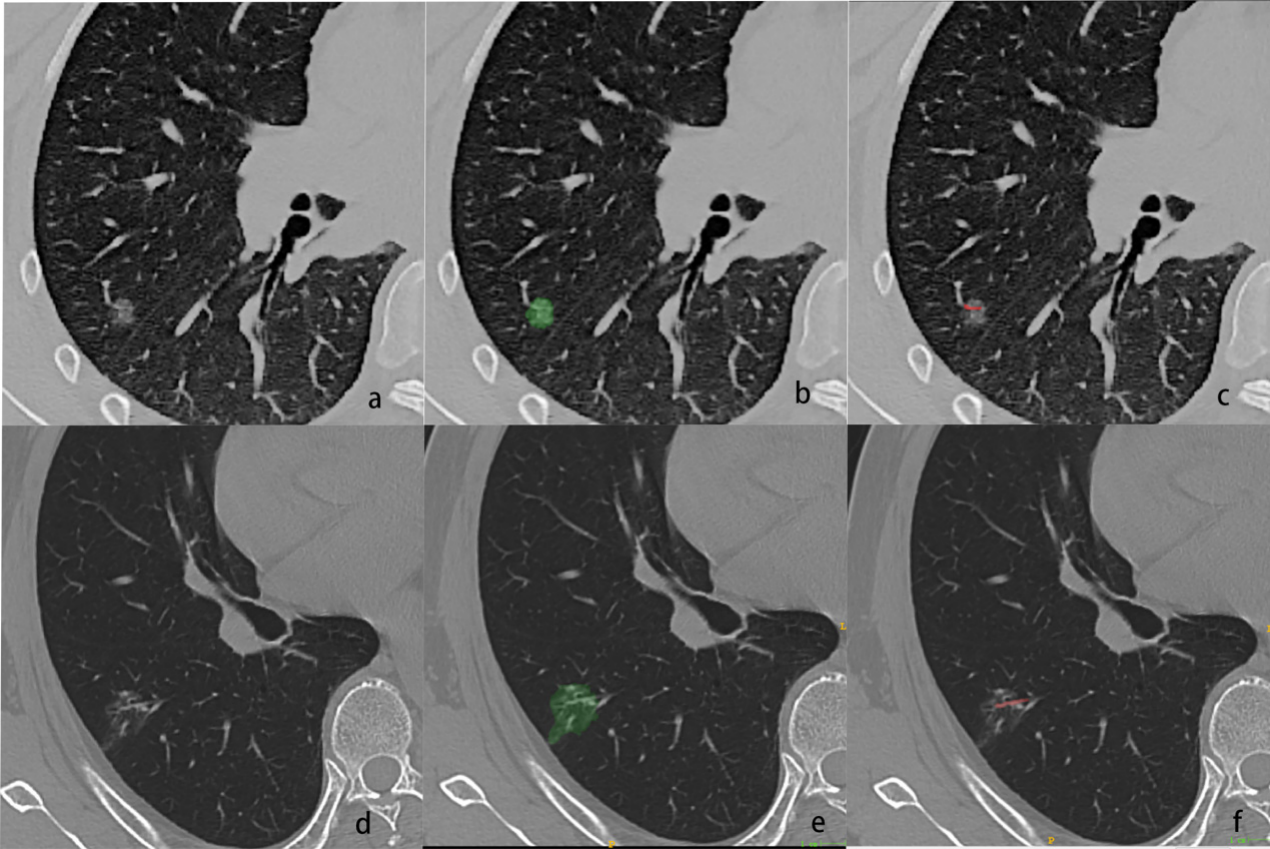
A 46-year-old woman with AIS presented as a pGGN. The nodule had a long diameter of approximately 0.7 cm and a short diameter of approximately 0.5 cm, with an average CT value of −524 HU. (**a**) CT showing the nodule with a regular shape, without lobulation, spiculation, or pleural retraction. (**b**) The ROI of the largest cross-section of the nodule. (**c**) The ROI of the internal vascular structures within the nodule. (**d**–**f**) A 68-year-old man with MIA presented as an mGGN. The nodule had a long diameter of approximately 1.4 cm and a short diameter of approximately 0.7 cm, with an average CT value of −422 HU. (**d**) CT showing the nodule with an irregular shape, lobulation, and pleural retraction but no spiculation. (**e**) The ROI of the largest cross-section of the nodule. (**f**) The ROI of the internal vascular structures within the nodule. **Abbreviations:** GGN, ground-glass nodule; CT, computed tomography; AIS, adenocarcinoma in situ; MIA, minimally invasive adenocarcinoma; mGGN, mixed GGN; pGGN, pure GGN; ROI, region of interest.

Tumor segmentation: All GGN CT images were imported into ITK-SNAP (version 3.6.0; www.itk-snap.org) for segmentation. Two physicians, one with 9 years and one with 10 years of experience in chest imaging for disease diagnosis, independently delineated the edges of the largest cross-sectional area of the lesions and internal vascular structures. Regions of interests (ROIs) were created based on pixel points and designated as ROI1 (lesion) and ROI2 (internal vessel), which were then saved in NII format (Fig. **2**). In cases of disagreement during segmentation, consensus was reached through discussion and consultation.

**Fig. (3):**
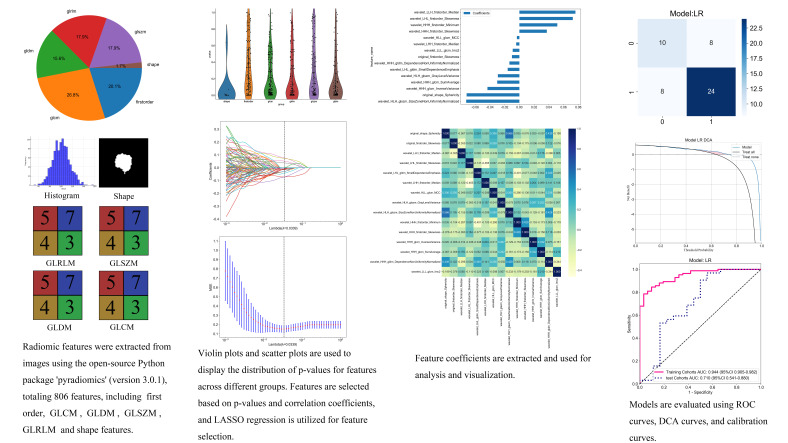
Radiomics workflow of this study, including feature extraction, selection, and model construction.

Feature extraction: Radiomics features were extracted using the open-source Python package PyRadiomics (version 3.0.1), with images normalized before feature computation. In total, 806 radiomics features were extracted, which were classified into feature-based (shape parameters, first-order texture parameters, gray-level run-length matrix, gray-level co-occurrence matrix, and gray-level size zone matrix) and filter-based (wavelet transforms, logarithmic features, and Laplacian operators) categories. The extracted feature data were then linked to the corresponding labels of lung nodule invasiveness, forming a complete research dataset (Fig. **3**).

Feature normalization: To account for varying scales in the original feature data, Z-score normalization was applied, standardizing all features by setting the mean to 0 and standard deviation to 1. This process minimized the impact of different scales on the model and ensured comparability during training. Statistical analyses, including the construction of feature distribution pie charts and selection of *P*-value, were conducted to identify features significantly associated with nodule invasiveness, forming the basis for subsequent feature selection and model training.

Feature selection: To reduce model complexity and enhance prediction accuracy, Pearson’s correlation coefficients and least absolute shrinkage and selection operator (LASSO) regression were employed for feature selection. Initially, Pearson’s correlation coefficient was used to evaluate the strength of linear relationships between each radiomics feature and lung nodule invasiveness. Values close to 1 or −1 indicated strong positive or negative correlations, respectively, whereas those close to 0 indicated no significant linear relationship. Features with an absolute correlation coefficient above a predefined threshold (*e.g.*, |r| > 0.3) were selected for dimension reduction in subsequent LASSO regression analyses. Through LASSO regression analysis, feature selection was further refined by incorporating an L1 regularization term in the loss function, compressing certain feature coefficients to zero, thereby achieving automatic feature selection and a sparse representation. Cross-validation was performed to determine the optimal regularization parameter λ, ensuring a balance between model bias and variance, ultimately identifying the most significant features for predicting lung nodule invasiveness (Fig. **3**). Penalties were imposed on the weights of nonessential features to prevent overfitting caused by redundant features. Synthetic minority oversampling was applied to the data, which significantly reduced the risk of overfitting.

#### 2.3.3 Model Construction and Evaluation

Patients were randomly categorized into training and validation sets in a 7:3 ratio. In the training set, features from different sequences and clinical characteristics were integrated early to form a comprehensive feature set. Based on the selected features, two machine learning models, logistic regression (LR) and support vector machine (SVM), were constructed, and cross-validation was carried out for model training and optimization. Both LR and SVM are widely used in predictive modeling, particularly in medical imaging. LR is effective for binary classification and provides clear interpretability of feature–outcome relationships, whereas SVM is well-suited for high-dimensional data and complex, nonlinear decision boundaries, making it ideal for radiomics studies with numerous features. To evaluate predictive performance, receiver operating characteristic curve analysis was performed, and key metrics, including area under the curve (AUC), accuracy, sensitivity, specificity, positive predictive value, and negative predictive value, were calculated. In addition, decision curve analysis (DCA), calibration curves, and DeLong’s test were applied to comprehensively assess the predictive performance of the clinical-radiomics-vascular model and clinical-radiomics-GGN model (Fig. **3**). It is necessary to strictly ensure the independence of the test set, and it should only be used for final model evaluation.

#### 2.3.4 Histopathological Analysis

Histopathological evaluation was conducted by a pathologist with 10 years of diagnostic experience. Upon collection, all samples were immediately fixed in 4% formaldehyde, embedded in paraffin blocks, and sectioned for subsequent staining analyses. Continuous 4-µm-thick sections were prepared from the paraffin-embedded samples. Standard hematoxylin-eosin staining was performed to examine the histological structure and cellular morphology of lung nodules, focusing on cell atypia and basement membrane penetration.

#### 2.3.5 Selection and Construction of Clinical Model Features

Using the “statsmodels” library in Python, all numerical features were standardized using the Z-score method to eliminate the impact of different feature scales. Univariate and multivariate regression analyses were performed using LR models, with the significance level set at α = 0.05 for all tests. Univariate regression analysis was performed to assess the relationship between each independent feature and nodule invasiveness, and statistically significant features were selected. Based on the obtained results, multivariate regression analysis was conducted to identify features with independent predictive value for nodule invasiveness. A stepwise selection method with a *P*-value threshold of 0.05 was used to iteratively filter features and construct the final multivariate regression model. Subsequently, the clinical prediction model for lung nodule invasiveness was developed by integrating clinical information and imaging features, primarily based on odds ratios, 95% confidence intervals (CIs), and *P*-values, to identify features with significant predictive capabilities.

#### 2.3.6 Statistical Analysis

All statistical analyses were performed using Statistical Package for the Social Sciences (SPSS) (version 22.0). Continuous variables with a normal distribution were expressed as means ± standard deviations, whereas those without a normal distribution were expressed as medians with interquartile ranges (IQRs). Pearson’s chi-square test was applied to categorical variables in the patients’ general clinical data and CT morphological features. For continuous variables, statistical test selection was based on distribution characteristics; an independent sample *t*-test was carried out for normally distributed data with homogeneity of variance, whereas the Wilcoxon rank-sum test was applied to data that did not meet these criteria. A *P*-value of <0.05 was considered statistically significant.

## 3 RESULTS

### 3.1 Clinical and Imaging Features

This study included 165 (59 males and 106 females) patients with GGNs. Of these, 126 presented with pGGNs, and 39 presented with mGGNs. The preinvasive group included 49 patients with AIS, whereas the invasive group included 116 patients with MIA and IA. In total, 12 clinical and imaging features were analyzed. The average age in the preinvasive group was 50 (IQR: 40–65) years, whereas that in the invasive group was 57 (IQR: 51–66) years, demonstrating a significant difference (Z = 2067.5, *P* = 0.006). The average CT values were −510.0 HU in the preinvasive group and −505.5 HU in the invasive group, but this difference was not statistically significant (Z = 2829.5, *P* = 0.966). The long diameter of GGNs was 0.939 ± 0.516 cm (mean ± standard deviation) in the preinvasive group and 1.128 ± 0.538 cm in the invasive group, showing a significant difference (t = −2.088, *P* = 0.038). Similarly, the average diameter of GGNs was 0.813 ± 0.436 cm in the preinvasive group and 0.972 ± 0.475 cm in the invasive group, exhibiting a significant difference (t = −2.009, *P* = 0.046) (Table **1**). However, no significant difference was observed in the short diameter of GGNs between the two groups (t = −1.815, *P* = 0.071). Regarding nodule type, pGGNs accounted for 89.8% of all GGNs in the preinvasive group compared with 70.7% in the invasive group, whereas mGGNs accounted for 10.2% of all GGNs in the preinvasive group compared with 29.3% in the invasive group, showing a significant difference (χ^2^ = 6.967, *P* = 0.008). In terms of morphology, the proportion of round/oval-shaped nodules was 91.8% in the preinvasive group and 79.3% in the invasive group, showing a significant difference (χ^2^ = 3.836, *P* = 0.049). In addition, a significant difference was observed in the presence of lobulation between the two groups (χ^2^ = 9.245, *P* = 0.002). However, no significant differences were observed between the two groups in terms of sex, nodule distribution, spiculation, and pleural traction (*P* = 0.865, 0.136, 0.192, and 0.088, respectively) (Table **1**).

### 3.2 Radiomics Models

#### 3.2.1 Predictive Value of the Radiomics-GGN Model

**Fig. (4):**
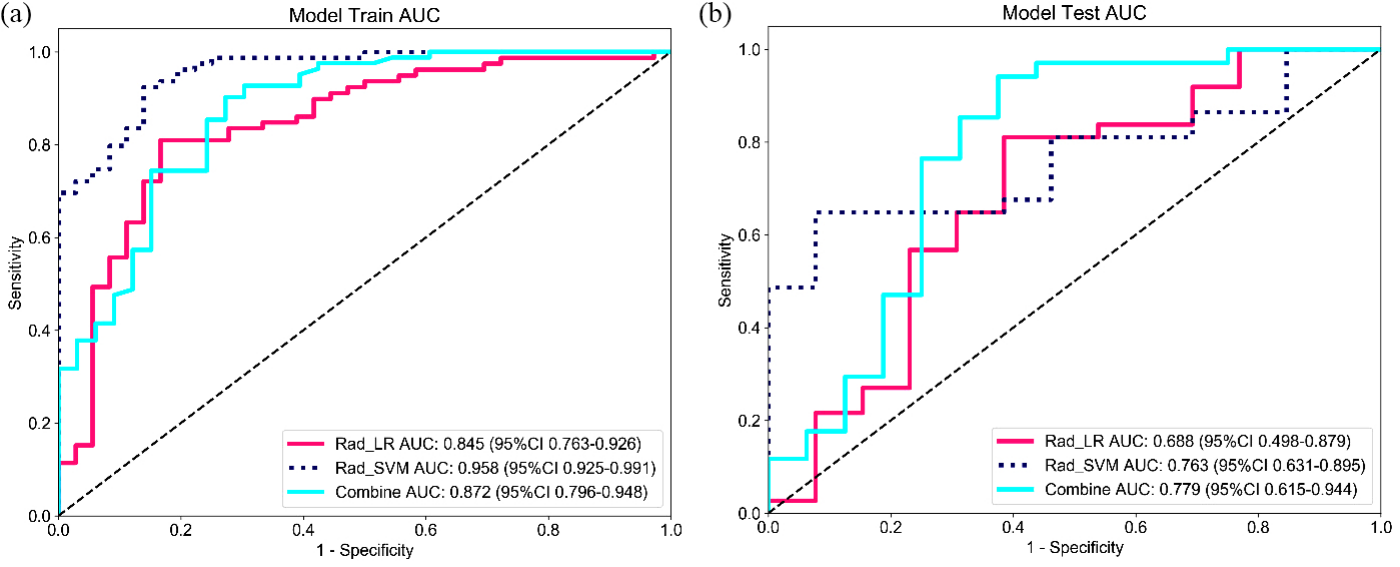
Predictive value of the radiomics-GGN model. (**a**, **b**) The SVM model outperformed the LR model in both sets. In addition, the clinical-radiomics model (combined) showed superior performance compared with models using radiomics alone.

**Fig. (5):**
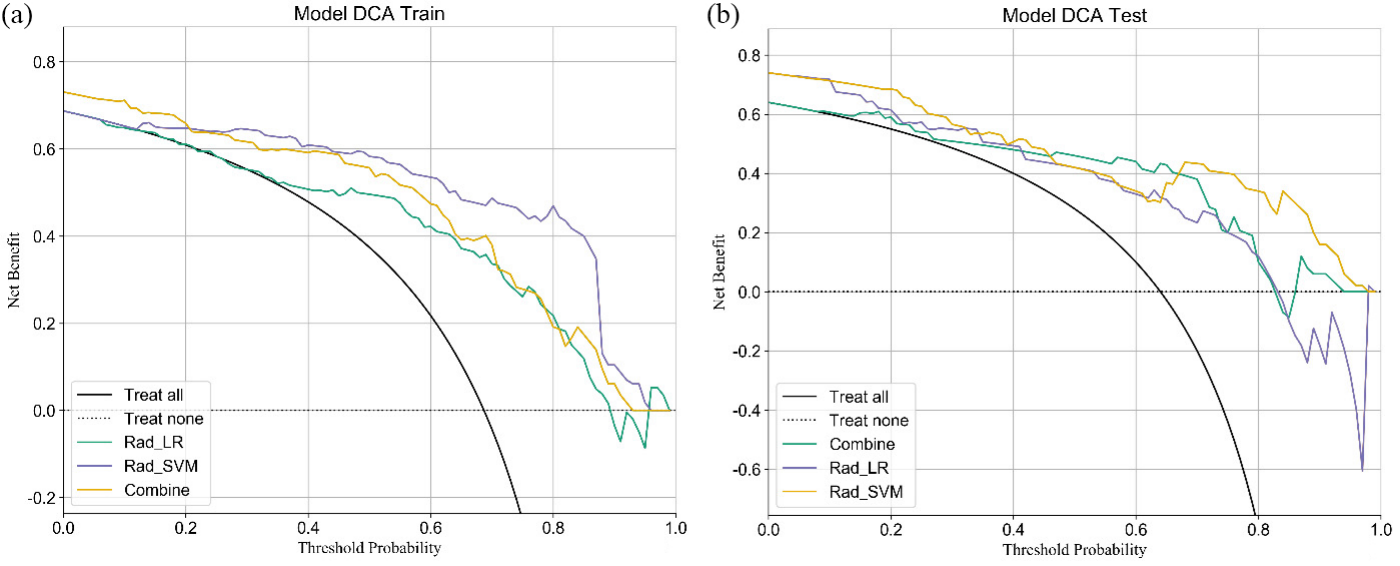
DCA curves for three models based on nodule ROIs in the training set (**a**) and validation set (**b**).

Patients were randomly categorized into the training and validation sets (7:3 ratio), with 81 invasive and 34 preinvasive cases in the training set and 35 invasive and 15 preinvasive cases in the validation set. A total of 806 radiomics features were extracted, and feature selection was performed using *P*-values, correlation coefficients, and LASSO regression, ultimately identifying 18 optimal features for model development. Both LR and SVM models were constructed. In the training set, the LR model achieved an accuracy of 0.809, with a sensitivity of 0.797, specificity of 0.833, and AUC of 0.845 (95% CI: 0.763–0.926). In the validation set, the LR model demonstrated an accuracy of 0.740, sensitivity of 0.784, specificity of 0.615, and AUC of 0.688 (95% CI: 0.498–0.879). The SVM model outperformed the LR model in the training set, achieving an accuracy of 0.896, sensitivity of 0.911, specificity of 0.861, and AUC of 0.958 (95% CI: 0.925–0.991). In the validation set, the SVM model achieved an accuracy of 0.700, with a sensitivity of 0.622, specificity of 0.923, and AUC of 0.763 (95% CI: 0.631–0.895) (Table **2** and Fig. **4**). DCA revealed that the radiomics model based on nodule ROIs provided a comprehensive and accurate data for clinical decision-making (Fig. **5**).

#### 3.2.2 Predictive Value of the Radiomics-vascular Model

**Fig. (6):**
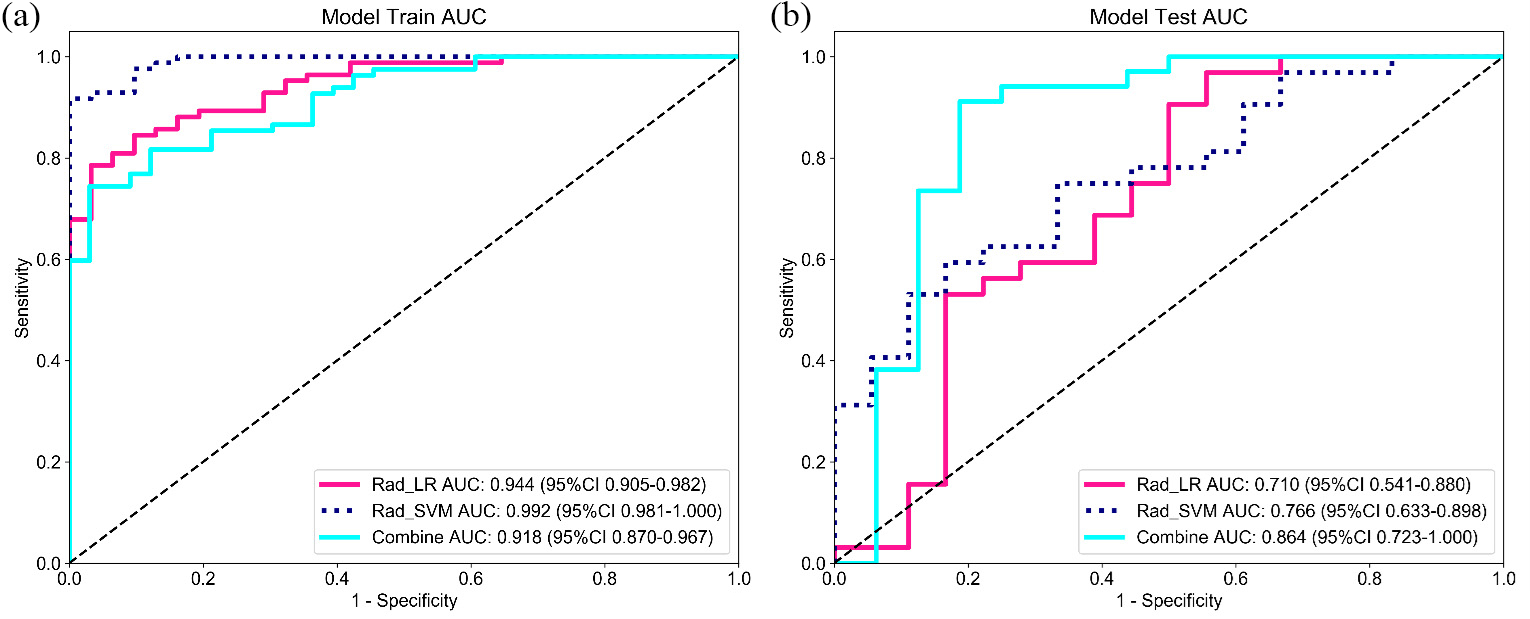
Predictive value of the radiomics-vascular model. (**a**, **b**) The SVM model outperformed the LR model in both sets. The clinical-radiomics model (combined) showed superior performance in the validation set.

**Fig. (7):**
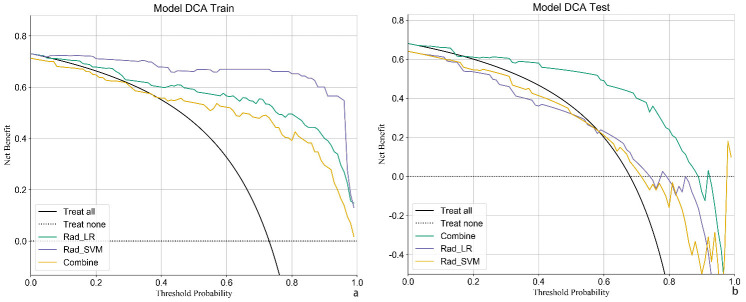
DCA curves for three models based on vascular features, both in the training set (**a**) and validation set (**b**).

Feature selection was conducted using *P*-values, correlation coefficients, and LASSO regression, ultimately identifying 15 optimal features for model development. Both LR and SVM models were constructed. In the training set, the LR model achieved an accuracy of 0.826, with an AUC of 0.944 (95% CI: 0.906–0.982), sensitivity of 0.774, and specificity of 0.968. In the validation set, the LR model demonstrated an accuracy of 0.76, with an AUC of 0.71 (95% CI: 0.541–0.880), sensitivity of 0.937, and specificity of 0.444 (Table **3** and Fig. **6**). In the training set, the SVM model exhibited better performance than the LR model, achieving an accuracy of 0.93, AUC of 0.992 (95% CI: 0.981–1.000), sensitivity of 0.905, and specificity of 1.000. However, in the validation set, the SVM model showed an accuracy of 0.66, with an AUC of 0.766 (95% CI: 0.633–0.899), sensitivity of 0.562, and specificity of 0.833 (Table **4** and Fig. **6**). DCA confirmed that the radiomics model based on vascular ROIs provided the comprehensive and accurate data for clinical decision-making (Fig. **7**).

#### 3.2.3 Clinical-radiomics Models

**Fig. (8):**
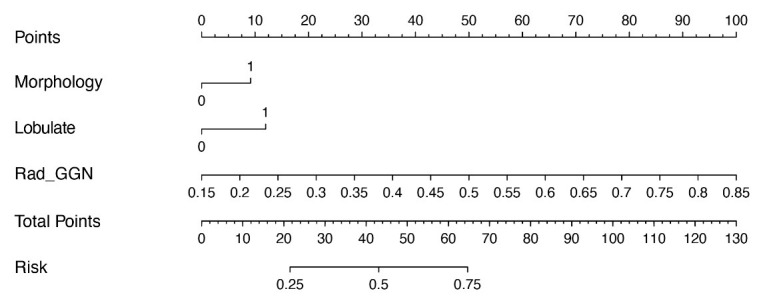
Nomogram constructed based on the clinical-radiomics-GGN model.

**Fig. (9):**
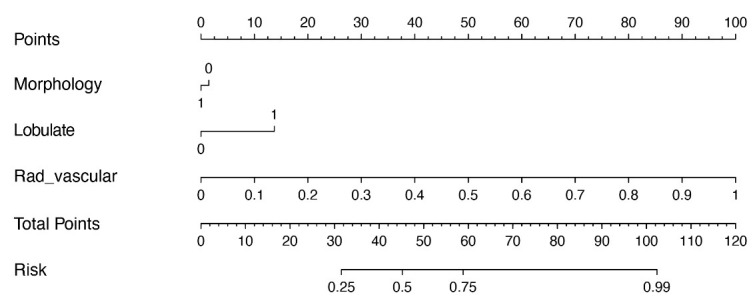
Nomogram constructed based on the clinical-radiomics-vascular model.

During the development of clinical models, LR analysis was performed. Univariate and multivariate LR analyses identified morphology and lobulation as independent risk factors for lung nodule invasiveness (*P* < 0.05) (Table **4**). These features were integrated during the development of clinical-radiomics models. Two models were constructed: clinical-radiomics-GGN (Fig. **8**) and clinical-radiomics-vascular (Fig. **9**).

#### 3.2.4 Predictive Value of the Clinical-radiomics-GGN model for GGN Invasiveness

The clinical-radiomics-GGN model was developed by integrating the radiomics features of GGNs with clinical imaging characteristics. Performance analysis revealed that in the training set, the model achieved an accuracy of 0.843, sensitivity of 0.890, and specificity of 0.727, with an AUC of 0.872 (95% CI: 0.796–0.948). In addition, both the precision and F1 scores were 0.89. In the validation set, the model maintained strong predictive performance, achieving an accuracy of 0.820, sensitivity of 0.912, and specificity of 0.625, with an AUC of 0.779 (95% CI: 0.615–0.944) (Table **5** and Fig. **4**). These metrics indicated that the model maintained high predictive accuracy and reliability in the validation set. Notably, the clinical-radiomics model outperformed models based on only radiomics features, highlighting the additional value of incorporating clinical imaging characteristics.

#### 3.2.5 Predictive Value of the Clinical-radiomics-vascular Model for GGN Invasiveness

The clinical-radiomics-vascular model was developed by integrating radiomics features of vascular ROIs within the nodules with clinical imaging characteristics. In the training set, the model achieved an accuracy of 0.800, sensitivity of 0.732, and specificity of 0.970, with an AUC of 0.918 (95% CI: 0.870–0.967). The precision and F1 scores were 0.984 and 0.839, respectively. In the validation set, the model demonstrated strong predictive performance, with an accuracy of 0.860, sensitivity of 0.882, specificity of 0.812, and AUC of 0.864 (95% CI: 0.723–1.000) (Table **5** and Fig. **6**). The clinical-radiomics model based on vascular ROIs exhibited superior predictive efficacy compared with models based solely on radiomics features and outperformed the clinical-radiomics-GGN model based on nodule ROIs.

**Fig. (10):**
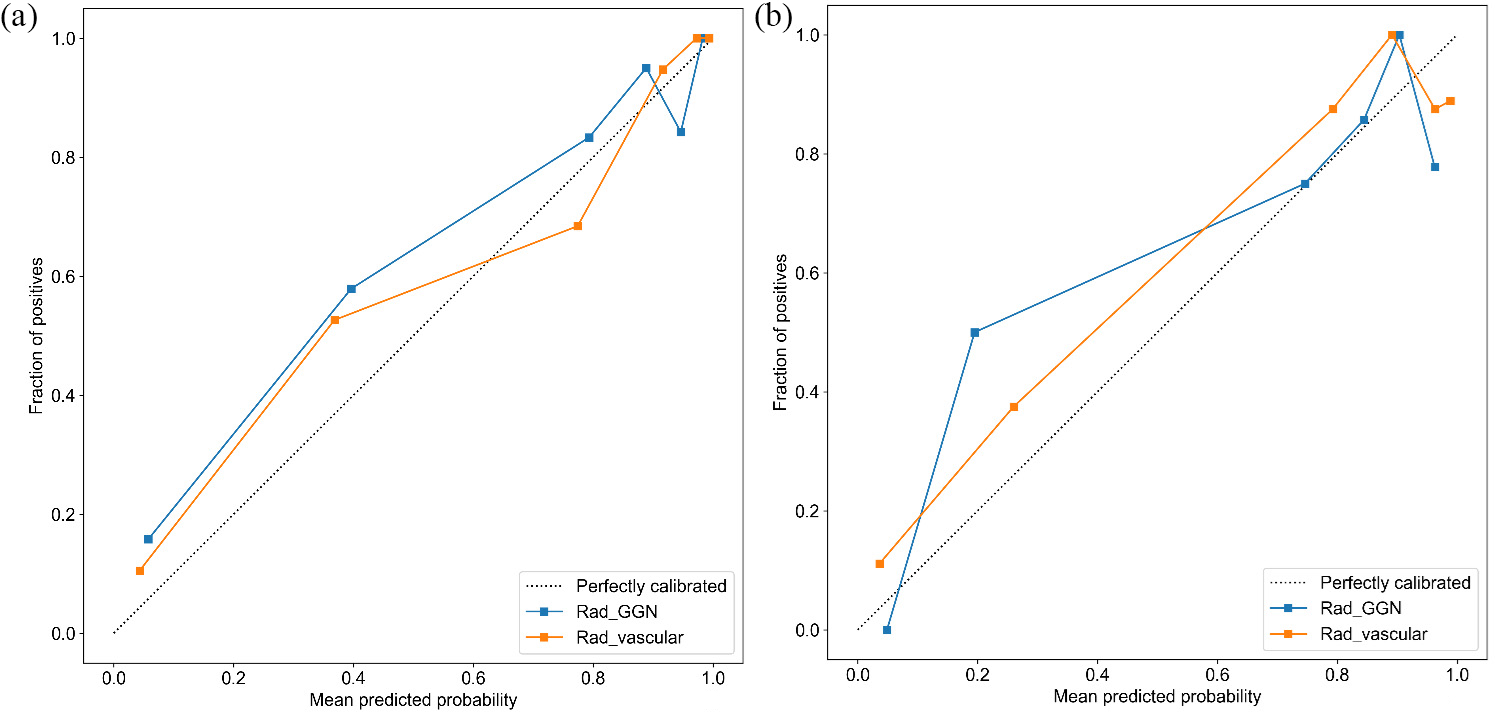
Calibration curves of the two clinical-radiomics models in the training set (**a**) and validation set (**b**). The results indicate that the clinical-radiomics model based on vascular ROIs (orange curve) demonstrated higher efficacy in clinical practice.

DCA and calibration curve analyses demonstrated that the clinical-radiomics model based on vascular ROIs showed higher efficacy than the clinical-radiomics-GGN model in clinical practice, with predicted outcomes aligning more closely with actual results (Figs. **7** and **10**). In addition, DeLong’s test revealed significant differences in predicting the invasiveness of pulmonary nodules between the clinical-radiomics-vascular model and the clinical-radiomics-GGN model, both in the training set (Z = 6.043, P < 0.01) and the validation set (Z = 3.170, P < 0.01), further confirming the superiority of the vascular-based model.

## 4 DISCUSSION

As age increases, the risk of tumor development rises, with lung cancer incidence substantially increasing after the age of 40 years [15]. The Fleischner Society Guidelines (2017) [16] report a higher malignancy rate for GGNs in the upper lobes. In our study, 111 nodules (including 27 preinvasive and 84 invasive nodules) were located in the upper lobes, possibly due to higher oxygen levels promoting tumor growth. The growth patterns of GGNs define their CT imaging characteristics. GGNs originate from adherent tumor cell growth, leading to alveolar epithelium thickening and reduced alveolar air content, appearing as pure ground-glass opacity on CT. Conversely, invasive areas follow nonadherent growth patterns (acinar, papillary, micropapillary, and solid), further reducing alveolar air content and increasing CT density or solid components. Ichinose *et al.* [17] identified high CT values as a key predictor of histological invasiveness in GGNs. Density types differed significantly between groups (*P* = 0.008), while average CT values showed no significant difference. This could be attributed to minimal solid components in some GGNs, which did not significantly affect the overall CT values. Spiculation, a strong malignancy indicator, reflects fibroblast proliferation, fibrous contraction, and tumor invasion into surrounding tissue, appearing as fine, needle-like projections on CT. In our study, spiculation did not significantly distinguish preinvasive from invasive lesions, likely due to the small size and early stage of tumors lacking matrix invasion. In addition, pleural retraction is often associated with GGN invasiveness, likely due to invasive lesions exerting traction on the pleura *via* the pulmonary fibrous framework. In our study, pleural indentation was more common in invasive lesions, but the difference was not significant (*P* = 0.088). We measured the maximum and perpendicular diameters at the axial maximum level, using their average value as the mean diameter. Invasive GGNs had significantly larger and average diameters than preinvasive ones, aligning with prior studies [18, 19]. Liu *et al.* [18] analyzed 105 GGNs and reported a critical diameter of 12.55 mm for invasiveness, whereas Zhou *et al.* [19] suggested 13.6 mm as the critical diameter, with variations likely due to different measurement techniques. Currently, no standardized threshold exists for GGN invasiveness. Irregular morphology and lobulation were more common in invasive GGNs, with multivariate analysis identifying irregular morphology as an independent predictor of invasiveness. Pathologically, differential growth rates and interlobular septa may result in surface irregularities and lobulation on CT. A previous study showed that lobulation is commonly observed in lung cancer [20].

Since the introduction of radiomics by Lambin *et al.* [11] in 2012, it has been widely integrated into medical imaging and pathology, particularly for assessing tumor heterogeneity. Radiomics is commonly used for the diagnosis, staging, grading, and evaluation of various malignant diseases, as well as for assessing therapeutic effects and prognosis [21-23]. Romeo *et al.* [24] and Shen *et al.* [25] demonstrated that radiomics classifiers and combined models could effectively distinguish the nature of lung nodules. Several studies [26-28] have also confirmed the diagnostic value of radiomics in predicting GGN invasiveness. For instance, Jiang *et al.* [29, 30] and Wu *et al.* [31] used radiomics to evaluate specific signs and peritumoral features of GGNs, further supporting their predictive value. However, studies on the radiomics features of internal vascular structures within GGNs remain limited. In our study, radiomics features were extracted from entire GGNs and their internal vascular structures using various algorithms. Subsequently, relevant features were selected and prediction models were developed. These models were then combined with traditional CT signs to enhance their predictive accuracy. Among them, the model based on internal vascular ROIs exhibited the best diagnostic performance in the training set. Vascular density and complexity in GGNs correlate with malignancy, as increased angiogenesis often indicates greater invasiveness [32]. Liang *et al.* [32] demonstrated that analysis of vascular features can effectively differentiate invasive nodules from noninvasive ones. The diagnostic value of internal vascular structures in pulmonary nodules can be understood through several key aspects, such as (i) vascular density and nodule nature: increased vascular density within a nodule suggests active metabolism and growth, indicating malignancy, as tumors require robust angiogenesis for nutrient and oxygen supply; (ii) vascular morphological features: irregular vascular structures, branching patterns, and permeability are critical for determining malignancy. Malignant nodules typically exhibit complex, irregular vascular networks, which are detectable *via* high-precision imaging; and (iii) angiogenesis and invasiveness: the degree of vascular formation within nodules is closely linked to their invasive potential. Invasive lung cancer often exhibits marked neovascularization, promoting tumor growth, invasion, and metastasis, with vascular abnormalities becoming more apparent as the disease progresses. Gao *et al.* [14] suggested that vascular anomalies are more frequently observed in MIA- and IA-classified GGNs. In clinical practice, the assessment of fine vessels within GGNs is often subjective. Advances in imaging have made radiomics a powerful tool for extracting detailed vascular features beyond basic morphology, including complexity, uniformity, and spatial relationships. Quantifying vascular morphology provides a noninvasive, objective approach to differentiate benign from malignant nodules. Moreover, previous studies [33-35] demonstrated that combining radiomics with traditional CT features significantly improves the model’s predictive accuracy for GGN invasiveness. Our study shows that combining radiomics with clinical features enhances predictive performance, underscoring the value of vascular radiomics in lung cancer assessment. Given the model’s potential clinical application, future studies should include external multicenter validation to confirm its generalizability. Differences in imaging protocols and patient populations across institutions may affect radiomic feature robustness. Therefore, multicenter studies are needed to ensure model reliability and support broader clinical adoption.

Our study had some limitations. First, all samples were obtained from a single center and analyzed retrospectively, which may have introduced selection bias. Second, the single-center nature of our study and the lack of an external validation set may limit the generalizability of our findings. Although internal validation through classification into training and validation sets in a 7:3 ratio provided insights into the model’s performance, external validation with multicenter data is crucial for confirming robustness and clinical applicability. Third, the uneven distribution of GGN pathological subtypes in our dataset, with fewer PGLs, may have introduced bias. This discrepancy likely results from the fact that most PGLs require follow-up rather than surgical removal, leading to their underrepresentation in the study.

## CONCLUSION

Radiomics models offer a valuable tool for the preoperative prediction of GGN invasiveness, with models based on radiomics features of internal vascular structures demonstrating superior predictive efficiency. For radiologists, this model serves as a noninvasive decision-support tool, aiding in the differentiation between high-risk GGNs that require early intervention and low-risk nodules suitable for conservative management. By enabling the noninvasive assessment of GGN invasiveness, this model can reduce unnecessary biopsies in low-risk cases. With continuous advancements in medical imaging technology and data analysis methods, radiomics is expected to play an increasingly significant role in the diagnosis, treatment, and management of lung nodules and other pulmonary diseases.

## Figures and Tables

**Table 1:** Comparison of clinical and imaging features between the invasive and preinvasive groups.

**Parameters**		**Group**		**Statistical Method**	**Test Value**	***P***
		**Pre-invasive Group** ** (n=49)**	**Invasive Group ** **(n=116)**			
Age (years)		50.0(40.0-65.0)	57.0(51.0-66.0)	Wilcoxon Rank-Sum test	2067.5	0.006 **
Gender	Female	31 (63.3%)	75 (64.7%)	Pearson's Chi-squared test	0.029	0.865
	Male	18 (36.7%)	41 (35.3%)			
CT Value (HU)		-510.000(-638.000--356.000)	-505.500(-598.250--412.000)	Wilcoxon Rank-Sum test	2829.5	0.966
Long Diameter (cm)		0.939±0.516	1.128±0.538	Independent Samples t-test	-2.088	0.038 *
Short Diameter (cm)		0.688±0.374	0.816±0.430	Independent Samples t-test	-1.815	0.071
Average Diameter (cm)		0.813±0.436	0.972±0.475	Independent Samples t-test	-2.009	0.046 *
Location	Right upper lung	20 (40.8%)	55 (47.4%)	Pearson's Chi-squared test	7.007	0.136
	Right lower lung	14 (28.6%)	15 (12.9%)			
	Right middle lung	2 (4.1%)	4 (3.4%)			
	Left upper lung	7 (14.3%)	29 (25.0%)			
	Left lower lung	6 (12.2%)	13 (11.2%)			
Shape	Round/Oval	45 (91.8%)	92 (79.3%)	Pearson's Chi-squared test	3.836	0.049*
	Irregular	4 (8.2%)	24 (20.7%)			
Lobulation	No	45 (91.8%)	81 (69.8%)	Pearson's Chi-squared test	9.245	0.002 **
	Yes	4 (8.2%)	35 (30.2%)			
Spiculation	No	32 (65.3%)	63 (54.3%)	Pearson's Chi-squared test	1.705	0.192
	Yes	17 (34.7%)	53 (45.7%)			
Pleural Traction	No	36 (73.5%)	69 (59.5%)	Pearson's Chi-squared test	2.912	0.088
	Yes	13 (26.5%)	47 (40.5%)			
Density	Pure ground glass	44 (89.8%)	82 (70.7%)	Pearson's Chi-squared test	6.967	0.008 **
	Mixed ground glass	5 (10.2%)	34 (29.3%)			

Values are given as n (%) or mean ± SD or median (interquartile range [IQR]). * indicates *P* < 0.05, ** indicates *P* < 0.01.

**Table 2:** Predictive value of the radiomics model based on nodule ROIs for GGN invasiveness.

**Model**	**Category**	**ACC**	**AUC (95%CI)**	**SEN**	**SPE**	**PPV**	**NPV**	**Precision**	**F1**
LR-radiomics-GGN model	Training set	0.809	0.845 (0.763 - 0.926)	0.797	0.833	0.913	0.652	0.913	0.851
	Validation set	0.740	0.688 (0.498 - 0.879)	0.784	0.615	0.853	0.500	0.853	0.817
SVM-radiomics-GGN model	Training set	0.896	0.958 (0.925 - 0.991)	0.911	0.861	0.935	0.816	0.935	0.923
	Validation set	0.700	0.763 (0.631 - 0.895)	0.622	0.923	0.958	0.462	0.958	0.754

**Table 3:** Predictive value of the radiomics-vascular model for GGN invasiveness.

**Model**	**Category**	**ACC**	**AUC (95%CI)**	**SEN**	**SPE**	**PPV**	**NPV**	**Precision**	**F1**
LR-radiomics- vascular model	Training set	0.826	0.944 (0.906 - 0.982)	0.774	0.968	0.985	0.612	0.985	0.867
	Validation set	0.760	0.710 (0.541 - 0.880)	0.937	0.444	0.750	0.800	0.750	0.833
SVM-radiomics- vascular model	Training set	0.930	0.992 (0.981 - 1.000)	0.905	1.000	1.000	0.795	1.000	0.950
	Validation set	0.660	0.766 (0.633 - 0.899)	0.562	0.833	0.857	0.517	0.857	0.679

LR, logistic regression; SVM, support vector machine; ACC, accuracy; AUC, area under the curve; CI, confidence interval; SEN, sensitivity; SPE, specificity; PPV, positive predictive value; NPV, negative predictive value.

**Table 4:** Logistic regression analysis of clinical and imaging features between the invasive and preinvasive groups.

**Parameters**	**Univariate Logistic Regression**		**Multivariate Logistic Regression**	
	**OR (95%CI)**	***P***	**OR (95%CI)**	***P***
Age	1.003 (0.997-1.008)	0.447	-	
Gender	0.941 (0.822-1.077)	0.454	-	
CT value	1.000 (0.999-1.000)	0.221	-	
Long diameter	0.924 (0.817-1.044)	0.285	-	
Short diameter	0.933 (0.795-1.096)	0.478	-	
Average diameter	0.923 (0.801-1.064)	0.351	-	
Location	0.998 (0.956-1.042)	0.933	-	
Shape	1.356 (1.147-1.605)	0.003**	1.289 (1.083-1.534)	0.017*
Lobulation	1.249 (1.075-1.451)	0.015*	1.173 (1.005-1.368)	0.049*
Spiculation	1.152 (1.012-1.311)	0.073	-	
Pleural traction	1.031 (0.901-1.181)	0.704	-	
Density	1.191 (1.023-1.387)	0.059	-	

**Table 5:** Predictive value of different clinical-radiomics models for GGN invasiveness.

**Model**	**Category**	**ACC**	**AUC (95%CI)**	**SEN**	**SPE**	**PPV**	**NPV**	**Precision**	**F1**
clinical-radiomics -GGN model	Training set	0.843	0.872 (0.796-0.948)	0.890	0.727	0.890	0.727	0.890	0.890
	Validation set	0.820	0.779 (0.615-0.944)	0.912	0.625	0.838	0.769	0.838	0.873
clinical-radiomics -vascular model	Training set	0.800	0.918 (0.870-0.967)	0.732	0.970	0.984	0.593	0.984	0.839
	Validation set	0.860	0.864 (0.723-1.000)	0.882	0.812	0.909	0.765	0.909	0.896

ACC, accuracy; AUC, area under the curve; CI, confidence interval; SEN, sensitivity; SPE, specificity; PPV, positive predictive value; NPV, negative predictive value.

DeLong’s test revealed significant differences between the two clinical-radiomics models in both the training (Z = 6.043, *P* < 0.01) and validation (Z = 3.170, *P* < 0.01) sets.

## Data Availability

All data generated or analyzed during this study are included within the article.

## LIST OF ABBREVIATIONS

GGN: Ground-Glass Nodule
AIS: Adenocarcinoma *In Situ*
PGL: Precursor Glandular Lesions
MIA: Minimally Invasive Adenocarcinoma
IA: Invasive Adenocarcinoma
mGGN: Mixed GGN
pGGN: Pure GGN
WHO: World Health Organization
CT: Computed Tomography
LD-CT: Low-Dose Computed Tomography
ROI: Region Of Interest
LASSO: Least Absolute Shrinkage And Selection Operator
LR: Logistic Regression
SMOTE: Synthetic Minority Oversampling Technique
ACC: Accuracy
SEN: Sensitivity
SPE: Specificity
SVM: Support Vector Machine
AUC: Area Under the Curve
DCA: Decision Curve Analysis
CI: Confidence Interval
IQR: Interquartile Range

## STANDARDS OF REPORTING

STROBE guidelines were followed.
